# Changes in fatty acid levels after consumption of a novel docosahexaenoic supplement from algae: a crossover randomized controlled trial in omnivorous, lacto-ovo vegetarians and vegans

**DOI:** 10.1007/s00394-022-03050-3

**Published:** 2022-11-23

**Authors:** Elena García-Maldonado, Alexandra Alcorta, Belén Zapatera, M. Pilar Vaquero

**Affiliations:** Department of Metabolism and Nutrition, Institute of Food Science, Technology and Nutrition (ICTAN), CSIC, José Antonio Novais 10, 28040 Madrid, Spain

**Keywords:** Supplementation, Docosahexaenoic acid, Fatty acids, Omega-3, Omega-6, Vegetarian, Vegan

## Abstract

**Purpose:**

To determine serum fatty acids of lacto-ovo vegetarian (LOV), vegan (VEG) and omnivorous (OMN) adults, and to analyse the effects of consuming a docosahexaenoic acid (DHA) supplement of vegetable origin on fatty acid profile.

**Methods:**

A randomized, double-blind, placebo-controlled crossover design was conducted in healthy adults. Volunteers (*n* = 116) were randomly assigned to a DHA-supplement (dose 250 mg/day), made from the microalgae *Schizochytrium sp*., or a placebo during 5-week periods separated by a 5-week washout interim period. Compliance and dietary intake were estimated and serum fatty acids were determined by gas chromatography. Results were analysed by mixed linear models.

**Results:**

Percentage of linoleic acid (C18:2n6) in serum was the highest among the fatty acids in the three diet groups, followed by oleic (C18:1n9) and palmitic (C16:0) acids. Linoleic (C18:2n6) and alpha-linolenic (C18:3n3) acids were higher in VEG compared to OMN (*p* < 0.001), while in LOV, their levels were intermediate between the other groups. Women presented higher DHA (C22:6n3) than men (*p* < 0.001). The DHA-supplement increased serum DHA compared to placebo in the three diet groups (*p* < 0.001), and a higher increase was observed in VEG followed by LOV (*p* < 0.001). The ratio serum n-6/n-3 improved by the supplementation but remained higher in LOV and VEG than in OMN. In contrast, the DHA-supplement decreased docosapentaenoic (C22:5n3) and docosatetraenoic (C22:4n6) acids in all diet groups (*p* < 0.001) and increased the eicosapentaenoic to alpha-linolenic fatty acids ratio (*p* = 0.016).

**Conclusion:**

The DHA-supplement at dose of 250 mg/day was effective in increasing serum DHA either in omnivorous, lacto-ovo vegetarian and vegan adults.

**Clinical trial registration:**

Registered at Clinicaltrials.gov (www.clinicaltrials.gov), NCT04278482.

**Supplementary Information:**

The online version contains supplementary material available at 10.1007/s00394-022-03050-3.

## Introduction

Vegetarian diets are associated to low levels of the most relevant risk factors for chronic disease compared to omnivorous [1]. However, they may result in inadequate intake of some nutrients such as essential fatty acids. There are only two essential fatty acids: the polyunsaturated fatty acids (PUFA) linoleic acid (LA, C18:2n6) which belongs to the omega-6 family (n-6), and α-linolenic acid (ALA, C18:2n3) which belongs to the omega-3 family (n-3), with pro-inflammatory and anti-inflammatory properties, respectively. Indeed, both families must be balanced to maintain optimal health and metabolic functions. LA is a precursor of arachidonic acid (AA, C20:4n6), while ALA is a precursor of eicosapentaenoic acid (EPA, C20:5n3) and docosahexaenoic acid (DHA, C22:6n3). These long-chain PUFA n-3 play many important roles, as they are highly concentrated in brain and retinal phospholipids as well as being involved in neurological and cognitive developments. In addition, they exert anti-inflammatory and antithrombotic properties [2]. Nevertheless, the rate of conversion of ALA to EPA and DHA is very low in humans [3–5]. For this reason, it is advisable to ingest DHA directly, being fish its main dietary source.

Because vegetarian diets do not include fish and fish by-products, it is of great interest to have adequate sources of n-3 long-chain PUFA, and to know their bioavailability and metabolic fate. In this regard, there are many vegetable sources of the precursor ALA: flax-seeds, hemp seeds, chia seeds, nuts, rapeseed, soybean and their derived oils [6]. However, low serum levels of EPA and DHA have been reported in lacto-ovo vegetarians (LOV) and vegans (VEG) [6–8]. Our research group recently observed that only 10% of the vegetarians participating in a nutritional study consumed n-3 supplements, which was associated with higher serum EPA but no improvement on DHA [8].

In the search for a plant-based alternative to fish, seaweed culture has been developed. Microalgae, which in fact are eaten by fish and consequently they acquire the n-3 fatty acids, nowadays, represent the most promising DHA source for the vegetarian population. Specifically, *Schizochytrium sp*. oil shows high DHA concentrations [9]. Moreover, the use of this oil has been authorized by The European Commission as a novel food and food supplement to provide a maximum of 250 mg DHA/day for general population [10]. It is stated that an intake of 250 mg of DHA helps maintain normal brain function and normal vision. In addition, a combined intake of 250–500 mg/day of EPA and DHA is recommended in adults based on the prevention of cardiovascular risk [11, 12]. Much higher doses of these n-3 fatty acids have been used to obtain therapeutic effects (> 3 g/day) [13], however, when looking for the most suitable supplement for vegetarians, the non-fish source and the physiological/nutritional quantity are the main determining factors.

Several studies in humans have reported a significant increase in serum DHA after intake of algal oil supplements. Early studies on algal DHA supplementation were conducted using DHA at doses of 1.6 g/day in vegetarians and omnivorous [14] or in two groups of vegetarians, one of them consuming corn oil as control [15]. Increases in DHA in serum phospholipids and platelets were noticed after 3 and 6 weeks. Other works in postmenopausal women used DHA supplements at doses of 2.1 and 2.8 g/day and reported elevations in circulating DHA and decreases in plasma lipids indicative of reduced cardiovascular risk [16, 17]. However, these studies present some limitations such as the small sample size and the need to give several capsules per day to achieve the desired dose. Subsequently, Arterburn et al. [18] evaluated the bioavailability of DHA oils in capsules from two different algae (*Crypthecodinium cohnii* and *Schizochytrium sp*.) and found a dose-dependent response with doses from 200 to 1000 mg/day after 4 weeks, and Sarter et al. [19] analyzed the omega-3 index (DHA + EPA in erythrocytes) in a dry blood spot sample after supplementation with a vegetal oil containing 254 mg of DHA plus EPA for 4 months and found a clear increase. In these two works, the algal oil contained a mixture of DHA and EPA that should be taken into account in order to compare their results with others.

Therefore, in the present study, we hypothesised that a DHA-supplement from the algae *Schizochytrium sp*. has sufficient bioavailability at a dose compatible with the normal vegetarian diet, i.e. 250 mg/day. To date, randomized controlled trials on the efficacy of algal DHA at this dose in vegetarians are very scarce.

Accordingly, the aims of this study were to: (1) determine the fatty acid profile of vegetarians and omnivorous (OMN), (2) establish the possible differences due to the type of vegetarian diet, either including eggs and milk products (LOV) or excluding them (VEG), and (3) study the effects of consuming a DHA-supplement of vegetable origin on fatty acid profile of LOV, VEG and OMN. To this end, a randomized double-blind, placebo-controlled cross-over study was carried out in healthy Spanish participants.

## Methods

### Study population

The study was conducted at the Institute of Food Science, Technology and Nutrition (ICTAN-CSIC) according to the guidelines laid down in the Declaration of Helsinki. It was approved by the Ethics Committee of the Spanish National Research Council (CSIC), Madrid, Spain (2021/03/05, Ref. 046/2021) and the Ethics Committee for Clinical Research of the Hospital Puerta de Hierro-Majadahonda, Majadahonda, Spain (2021/03/08, Ref. PI176/19). It was registered in the Clinical Trials database (ClinicalTrials.gov ID: NCT04278482). Before the study began, participants received information about the study and the day they attended the first visit at the institute they signed an informed consent form expressing their agreement to participate in the study.

Participants were recruited via poster advertisements and a direct mailing sent to academic, social, health, and research institutions. The study was carried out between March and July of 2021 in Madrid, Spain. The inclusion criteria were: healthy adults aged 18–45, consumers of a vegetarian diet (for at least 6 months) or an omnivorous diet with low fish intake, below the Spanish current dietary recommendation of at least 2 servings/week [20]. Exclusion criteria were: minors (< 18 years) or adults over 45 years of age; pregnant women; consumers of fortified foods with n-3 fatty acids or phytosterols in the last 3 months; people with a diagnosed metabolic disease (liver disease, gastritis, irritable bowel, gastric ulcer, hypercholesterolemia, hypertension, diabetes); people with history of biliary colic, suffering from an eating disorder, or following any treatment that could modify the study variables. It was required not to have participated in any clinical study in the last 3 months.

### Study design and sample size calculation

The study had a randomized, double-blind, placebo-controlled crossover design with two 5-week periods separated by a 5 week interim washout period. In each period, to standardise the experimental conditions, one capsule (DHA-supplement or placebo) per day was taken by the participants with one of their main meals, because high DHA absorption is expected when consumed with meals [21].

The capsules were delivered in blisters packed in a white box and neither the participants nor the researchers knew the type of capsule given to each participant. The experimental period was established according to the recommendations of the human trials on lipids which was at least 4 weeks [7, 18, 22].

The volunteers were randomly assigned the DHA treatment or placebo with the Microsoft Excel randomization function (RAND). Participants were allocated to one sequence starting with DHA-supplement and then placebo, or starting with placebo and then DHA-supplement. However, there were a few cases were volunteers who lived together were provided with the same capsule type to avoid mixing capsules and improve adherence.

The statistical power was set at 85% and the confidence level at 95%. Taking serum DHA as the main variable and knowing its variability obtained in our previous study performed in similar vegetarians [8], a sample size of 76 volunteers was calculated to be sufficient to detect a difference of 20% in serum DHA. We increased this number to 90 to ensure that the statistical power would be sufficient even with dropouts once the clinical trial had started.

### Supplement and placebo capsules

The supplement was a vegetal DHA-supplement that contained 625 mg of total lipids from the microalgae *Schizochytrium sp*, of which 250 mg was DHA (Zamdeh Laboratories). The placebo was formulated to closely match the DHA-supplement in terms of total lipids and appearance. It contained 625 mg of olive oil. The capsules were given in blisters and neither participants nor researchers knew the type of capsule that was given to each participant. Participants had to take one capsule a day and swallow it whole. The composition of these capsules is shown on Electronic Supplementary Material (Tables S1 and S2).

### Health questionnaires and compliance control

Before the study started, volunteers were asked to fill in an on-line lifestyle and health questionnaire to monitor possible health problems and medication use and to check whether they met all the inclusion criteria and none of the exclusion criteria. In addition, volunteers completed a specific questionnaire during each intervention phase (weeks 5, 10, and 15) to monitor health status and capsule compliance.

The capsules were packaged in blister packs of 15 units each. The batch number was specified on each blister. Each volunteer received a total of 45 capsules of the assigned batch at the beginning of each period. Compliance was calculated by asking how many capsules were consumed during each intervention period and counting the number of capsules that the volunteers had to return at the end of the two periods.

### Dietary control

The volunteers were instructed to follow their usual diet and physical activity between visits. To monitor possible changes during the assay, at baseline and at 5, 10 and 15 weeks of the trial, all participants completed a 72 h detailed dietary intake report specifying the type and/or brand of the product and the portion or weight of food consumed. Dietary intake was analysed using the DIAL software (Alce Ingeniería, Madrid, Spain). This software allows the addition of new food products and their nutrient composition. Therefore, labelling information was used to complete the data for many foods, most of them formulated for vegans.

### Anthropometrical measurements, body composition, and blood pressure

At 0, 5, 10 and 15 weeks volunteers’ height, body weight, and waist and hip perimeters were measured, and body mass index (BMI) and body composition were obtained (Tanita BC-601, Tanita Ltd., Amsterdam, Netherlands). In addition, systolic and diastolic blood pressure was measured with a validated digital automated blood pressure monitor (IHealth KN-550BT, iHealthLabs Europe, Paris).

### Blood sampling and biochemical determinations

Blood samples were collected after a 10 h fasting period at weeks 0, 5, 10 and 15 of the study. Serum was isolated by centrifugation in a Jouan CR-312 centrifuge (Jouan Ltd, Ilkeston, UK) at 1000 g for 15 min and total cholesterol, HDL-cholesterol, LDL-cholesterol, triglycerides and glucose were analysed by autoanalyzer in fresh serum. Aliquots were stored at – 80 ºC for further fatty acid analysis.

### Serum fatty acid analysis

Fatty acids were analysed in serum following the technique by direct bimethylation according to Lee et al. [23]. Briefly, 300 µL of serum were lyophilized and 0.5 mL of internal standard C13:0 (0.1 mg/ml) dissolved in hexane was added prior to methylation. Fatty acid methyl esters were determined by gas chromatography (GC) using an Agilent 7820 A equipment (Agilent Technologies, CA, USA) with flame ionization detector. The analysis was carried out in split mode (split ratio 40:1) using an Agilent DB-23 capillary GC column (60 m × 250 μm × 0.25 μm) and helium as the carrier gas. Samples (1 μL) were injected using an automatic liquid sampler at a temperature of 50 °C, this was then ramped at 25 °C/min to 175 °C and then at 4 °C/min to 230 °C where it was held for 35.75 min. Data were expressed as percentage of total fatty acids (g/100 g). These analyses were performed in the Analysis Service Unit facilities of ICTAN and the Quality Management System comply with the requirements of UNE EN ISO 17025 and UNE EN ISO 9001:2015.

### Statistical analyses

Data were analysed using the Statistical Package for the Social Sciences (SPSS) for Windows version 26.0 (IBM SPSS Statistics for Windows Armonk, NY, USA) and all values are expressed as mean ± SEM, unless otherwise specified.

Two generalized mixed linear models were used to analyse the inter-individual differences due to dietary group and those intra-individual due to treatment. In the first model (Model 1), the fixed effects were: diet group (OMN, LOV, VEG), treatment (no-treatment, DHA-supplement, placebo), sex, and the interaction diet x treatment. Random effects were order of treatment (first placebo then DHA-supplement, first DHA-supplement then placebo) and participant (code). Repeated effect was visit (basal, after period 1, after washout, after period 2). Once minor differences were obtained between no-treatment and placebo with the first model, in the second model (Model 2), the same effects were analysed but only two levels of treatment were considered (DHA-supplement and placebo). To study the effects of diet group, sex and diet x sex on DHA changes, a two factors analysis of variance (ANOVA) was also employed. The significance level was set at *p* < 0.05.

## Results

### Participant recruitment

**Fig. 1:**
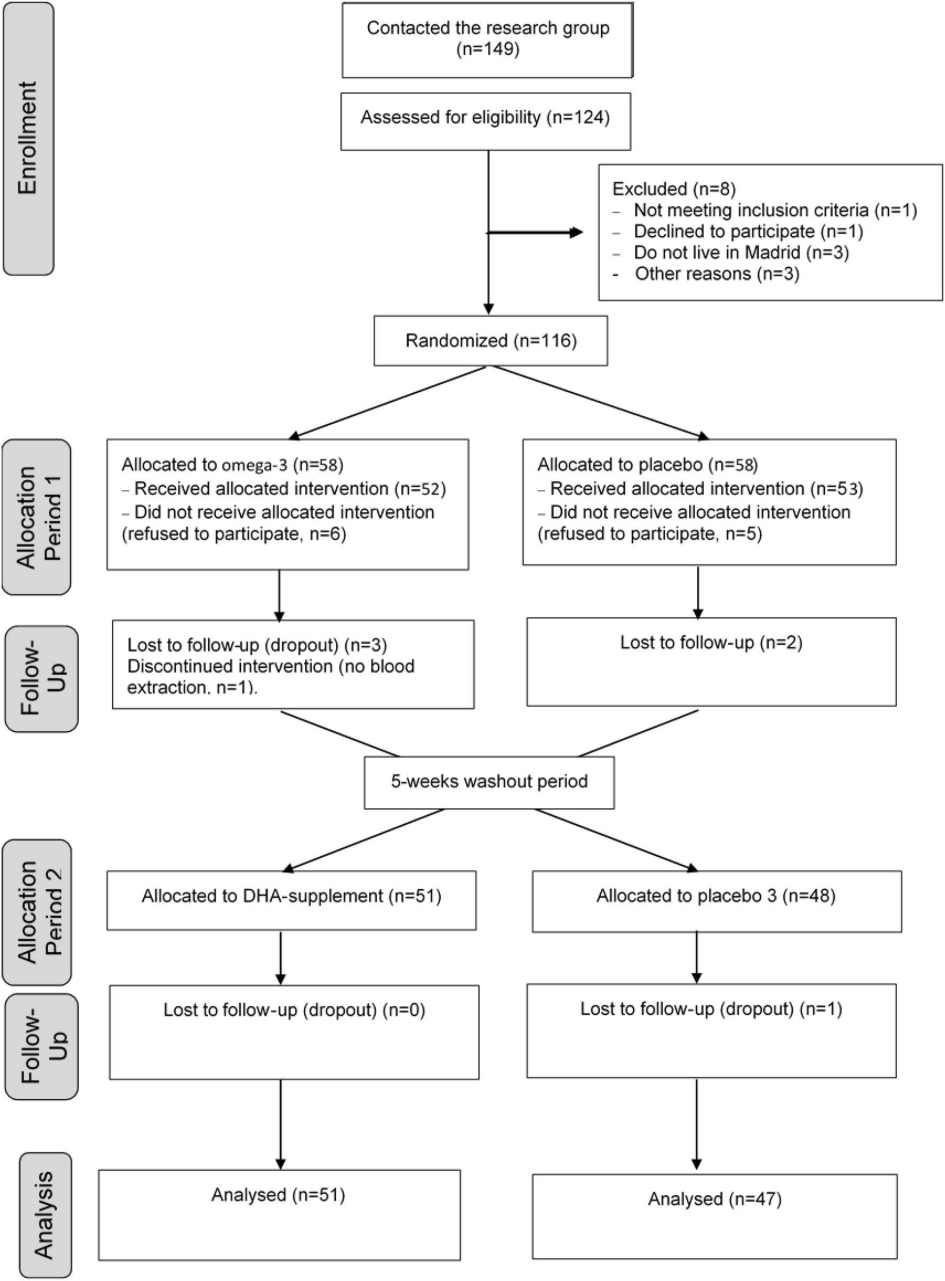
Consolidated Standards for Reporting Trials (CONSORT) flow diagram

The flow of participants is presented in the format of the CONSORT diagram (Fig. 1). From a total of 149 participants interested in the study, 124 were assessed for eligibility and 116 met all the inclusion criteria and none of the exclusion criteria. These were randomized to start with the DHA-supplement or the Placebo (*n* = 58 in each arm). Several participants did not attend the first visit and refused to participate, *n* = 6 participants did not receive the allocated DHA treatment and *n* = 5 did not receive the placebo. In the first period, there were three dropouts and one participant was without blood extraction in the DHA-supplement treatment, and there were two dropouts in the placebo. Therefore, there were *n* = 51 and *n* = 48 participants to receive the DHA-supplement and placebo, respectively, during the second period. One participant receiving placebo was lost to follow-up and finally the analysis of the second period was performed in *n* = 51 and *n* = 47 participants who received the DHA-supplement or the placebo, respectively. Total analysed was *n* = 98.

### Population characteristics

Participants were young adults, age 26.5 ± 0.3 years (mean ± SEM), apparently healthy, Caucasians (98%), 59% were women and the ratio omnivorous/vegetarian was 47/53%. The percentage of OMN, LOV and VEG of the total sample were, 43, 27 and 30%, respectively (Table 1). Referred to the order of treatment, 52 volunteers started to DHA-supplement and 53 volunteers started with placebo.

**Table 1:** Baseline characteristics of the participants by sequence of treatment and by total

Parameter	Total group	Order of treatment		*p*^*a*^
		DHA supplement-placebo	Placebo-DHA supplement	
Participants (*n*)	105	52	53	
Sex (*n*)				
Woman	62	33	29	
Men	43	19	24	
Diet (*n*)				
OMN	45	26	19	
LOV	28	14	14	
VEG	32	12	20	
Anthropometric determinations				
Age	25.88 ± 0.27	25.98 ± 0.598	25.65 ± 0.86	0.749
Weight (kg)	64.26 ± 0.58	62.57 ± 1.39	65.97 ± 1.70	0.124
Stature (m)	1.69 ± 0.41	1.69 ± 1.09	1.70 ± 1.24	0.646
BMI (kg/m^2^)	22.30 ± 0.15	21.72 ± 0.36	22.80 ± 0.51	0.087
Body fat (%)	20.65 ± 0.38	20.78 ± 1.07	21.65 ± 1.06	0.568
Muscle mass (kg)	48.37 ± 0.49	47.03 ± 1.18	48.36 ± 1.63	0.507
Abdominal perimeter (cm)	76.06 ± 0.44	74.76 ± 1.61	76.79 ± 1.30	0.246
Hip perimeter (cm)	95.23 ± 0.32	94.33 ± 0.91	95.76 ± 0.99	0.286
Blood pressure				
Systolic blood pressure (mmHg)	122.30 ± 0.57	124.17 ± 1.61	124.48 ± 1.40	0.884
Diastolic blood pressure (mmHg)	80.55 ± 0.44	81.98 ± 0.99	81.95 ± 1.12	0.984
Heart rate (lat/min)	74.46 ± 0.65	76.97 ± 2.00	76.55 ± 1.75	0.240
Biochemical determinations				
HDL-Cholesterol (mg/dL)	58.35 ± 0.79	62.79 ± 2.67	55.00 ± 1.98	**0.022**
LDL-Cholesterol (mg/dL)	87.97 ± 1.19	91.24 ± 3.19	84.26 ± 3.48	0.141
Total Cholesterol(mg/dL)	160.56 ± 1.52	167.67 ± 4.32	153.88 ± 4.44	**0.028**
Triglycerides (mg/dL)	70.51 ± 2.85	67.89 ± 3.18	73.34 ± 4.84	0.342
Glucose (mg/dL)	84.14 ± 0.37	82.20 ± 0.92	81.84 ± 0.96	0.785

Data are mean ± SEM

Bold values indicate that the significant differences

^a^Differences between order of treatment groups (independent samples *t* test)

Randomization produced two order groups that were not different in body composition, biochemical parameters, and dietary intake, except in HDL-cholesterol and total-cholesterol values that were significantly higher in the group with more OMN and more women (Table 1). Inter-participant differences due diet type and sex were observed (*p* < 0.001, split results not shown). Baseline percentiles of serum fatty acids according to diet OMN, LOV and VEG are presented on the supplementary Material (Table S3). The ratio n-6/n-3 and other ratios of fatty acids are also shown.

### Compliance and health status

Compliance was 97.1 ± 0.6% in period 1 and 96.4 ± 0.6% in period 2, without significant differences between periods (*p* = 0.364). Volunteers did not manifest any different discomfort consuming one type or another type of capsules. Due to compliance being high, the deviation from the protocol was negligible and the data of all volunteers were included in the analyses. Therefore, an “intention to treat” analysis was performed.

This clinical trial started after the third wave of COVID-19 in Spain, March 2021, and we recorded that due to possible infection/contagion several volunteers did not take the capsule on sporadic days. Other health issues were negligible and did not affect the trial performance.

### Dietary intake

Dietary intake was initially analysed using the mixed Model 1 and this showed significant differences due to diet type and gender, but the effects of treatment (no-treatment, DHA-supplement, placebo) and order of treatment (DHA-Placebo or Placebo-DHA) were not significant for any determination. Men ingested more energy and macronutrients than women (all *p* < 0.001) although for the specific intake of DHA and the ratio n-6/n-3 the gender differences were not significant.

Table 2 shows the energy and nutrient intakes of volunteers according to their diet, OMN, LOV or VEG, at baseline. It was observed that LOV and VEG ingested more carbohydrates than OMN (*p* = 0.002). The three diet groups had similar intake in terms of energy, proteins and total lipids, but consumption of SFA in VEG was significantly lower compared to OMN and LOV (*p* < 0.001). It was obtained a tendency to higher PUFA and higher LA (C18:2n6) in LOV and VEG compared to OMN (*p* = 0.057 and 0.067, respectively). LOV and VEG ingested less AA (C20:n6) than OMN, (*p* < 0.001). The intake of EPA and DHA by the two vegetarian groups was nearly null (both *p* < 0.001). However, LOV and VEG ingested more n-6 than OMN, although the differences were only significant in the case of VEG (*p* = 0.017), and consequently the ratio n-6/n-3 was higher in these two diet groups compared to OMN (*p* < 0.001).

**Table 2:** Dietary intake according to dietary groups

	OMN	LOV	VEG	*p*
Energy (kcal/day)	1961 ± 99	2124 ± 114	2095 ± 1103.71	0.494
Proteins (g/day)	87.50 ± 5.56	84.29 ± 7.99	75.42 ± 3.71	0.307
Carbohydrates (g/day)	185.09 ± 11.01	230.48 ± 10.18^a^	237.91 ± 12.80^a^	**0.002**
Lipids (g/day)	84.91 ± 4.64	85.14 ± 6.57	80.39 ± 7.26	0.827
SFA (g/day)	26.40 ± 1.67	23.19 ± 2.10	15.36 ± 1.41^a, b^	** < 0.001**
MUFA(g/day)	34.71 ± 2.48	30.80 ± 3.42	31.48 ± 3.44	0.604
PUFA (g/day)	12.00 ± 0.95	13.91 ± 1.38	18.86 ± 3.44	0.057
LA (g/day)	9.32 ± 0.89	11.77 ± 1.22	12.89 ± 1.40	0.067
ALA (g/day)	1.27 ± 0.13	1.55 ± 0.23	1.38 ± 0.20	0.559
AA (g/day)	0.16 ± 0.02	0.06 ± 0.02^a^	0.00 ± 0.00^a^	** < 0.001**
OA (g/day)	32.04 ± 2.40	27.26 ± 3.17	26.45 ± 2.94	0.277
EPA (g/day)	0.20 ± 0.06	0.00 ± 0.00^a^	0.00 ± 0.00^a^	** < 0.001**
DHA (g/day)	0.49 ± 0.12	0.08 ± 0.03^a^	0.00 ± 0.00^a^	** < 0.001**
n-3 (g/day)	2.03 ± 0.22	1.63 ± 0.23	1.38 ± 0.20	0.113
n-6 (g/day)	9.48 ± 0.89	11.83 ± 1.23	12.90 ± 1.40^a^	**0.017**
Ratio n-6/n-3	5.84 ± 0.46	9.06 ± 0.97^a^	10.77 ± 0.80^a^	** < 0.001**

Values are expressed in mean ± SEM and are calculated from data of baseline. Significant differences between diet groups (*p* < 0.05) by ANOVA

Bold values indicate that the significant differences

*OMN* omnivorous, *LOV* lacto-ovo vegetarian, *VEG* vegan

^a^Denotes significant differences compared to OMN

^b^Denotes significant differences compared to LOV

Table 3 presents dietary intake after placebo and DHA-supplement according to dietary group using the Model 2 for statistical analyses. The effect of treatment was not significant for any variable and the observed significant differences were all due to the diet type factor (Table 3).

**Table 3:** Dietary intake according to treatment and dietary group

	Placebo			DHA-supplement			*p*_D_
	OMN	LOV	VEG	OMN	LOV	VEG	
Energy (kcal/day)	1946 ± 100	2116 ± 175	1997 ± 75	2028 ± 97	2294 ± 146	1973 ± 88	**0.015**
Proteins (g/day)	93.00 ± 6.80	84.62 ± 9.70	72.90 ± 3.99	89.48 ± 6.31	86.28 ± 6.90	76.46 ± 5.74	**0.039**
Carbohydrates (g/day)	180.81 ± 12.50	223.85 ± 17.35^b^	232.81 ± 10.98^b^	188.98 ± 11.50	241.63 ± 18.44^b^	240.53 ± 12.81^b^	** < 0.001**
Lipids (g/day)	81.32 ± 4.71	88.88 ± 9.31	74.20 ± 4.68	91.82 ± 5.22	96.81 ± 8.30	67.62 ± 4.79	**0.001**
SFA (g/day)	25.10 ± 1.61	25.23 ± 3.16	14.36 ± 1.05^b, c^	29.83 ± 1.97	31.47 ± 3.91	13.90 ± 1.41^b, c^	** < 0.001**
MUFA(g/day)	33.52 ± 2.10	34.54 ± 3.63	29.94 ± 3.10	39.21 ± 2.60	37.24 ± 3.34	27.29 ± 2.72^b^	**0.006**
PUFA (g/day)	12.26 ± 1.15	16.21 ± 3.02	16.37 ± 1.36	11.63 ± 0.75	13.88 ± 1.85	14.47 ± 1.34	**0.010**
LA (g/day)	10.05 ± 0.96	13.81 ± 2.62	14.82 ± 1.22	9.71 ± 0.66	11.71 ± 1.59	13.65 ± 1.25	** < 0.001**
ALA (g/day)	1.16 ± 0.12	1.78 ± 0.41	1.85 ± 0.25	1.06 ± 0.09	1.54 ± 0.28	1.61 ± 0.23	**0.003**
AA (g/day)	0.21 ± 0.04	0.06 ± 0.02^b^	0.01 ± 0.00^b^	0.17 ± 0.02	0.10 ± 0.03^b^	0.02 ± 0.01^b, c^	** < 0.001**
OA (g/day)	30.85 ± 1.98	31.70 ± 3.47	27.87 ± 3.25	36.07 ± 2.47	32.32 ± 3.09	24.82 ± 2.57^b^	**0.013**
DHA (g/day)^a^	0.44 ± 0.11	0.05 ± 0.01^b^	0.01 ± 0.00^b^	0.21 ± 0.03	0.13 ± 0.04	0.00 ± 0.00^b, c^	** < 0.001**
EPA (g/day)	0.18 ± 0.05	0.00 ± 0.00^b^	0.00 ± 0.00^b^	0.04 ± 0.01	0.00 ± 0.00^b^	0.00 ± 0.00^b^	** < 0.001**
n-3 (g/day)	1.86 ± 0.23	1.85 ± 0.42	1.85 ± 0.25	1.35 ± 0.12	1.68 ± 0.29	1.61 ± 0.23	0.641
n-6 (g/day)	10.25 ± 0.97	13.88 ± 2.62	14.83 ± 1.22	9.88 ± 0.66	11.81 ± 1.59	13.67 ± 1.25^b^	**0.001**
Ratio n6/n3	6.95 ± 0.60	9.06 ± 0.63	11.79 ± 1.57^b^	7.96 ± 0.49	8.26 ± 0.78	11.95 ± 1.69^b^	** < 0.001**

Values are expressed in mean ± SEM

Bold values indicate that the significant differences

*OMN* omnivorous, *LOV* lacto-ovo vegetarian, *VEG* vegan

^a^The values of DHA are calculated only from food excluding the test supplement. Within placebo or treatment

^b^Denotes significant differences compared to OMN

^c^Compared to LOV (at least *p* < 0.05). The effects of treatment and interaction diet group x treatment were no significant

### Influence of diet type and DHA-supplementation on serum fatty acid percentages

There were marked differences due to diet type and treatment in serum fatty acids and no significant effect of the random factor order of treatment was observed. Using Model 1, it was shown that serum PAL was significantly higher and serum DHA significantly lower after placebo compared with no-treatment in VEG (*p* = 0.039 and *p* = 0.045, respectively, data not shown). No other significant difference between the no-treatment condition and placebo was detected. Women showed higher total SAT (*p* = 0.048) and PUFA (*p* = 0.026) while lower MUFA (*p* < 0.001) than men. Concerning the main variable of the study, the concentrations of DHA and ratios DHA/AA, DHA/ALA and DHA/EPA were higher in women than men (all, *p* < 0.001, data not shown).

Table 4 presents the results of the influence of the DHA-supplement compared to placebo in serum fatty acid percentages, for the three diet groups (Model 2). With regard to diet effects, the saturated PTC (C15:0) and PAL (C16:0) were lower in VEG compared to OMN and LOV (both *p* < 0.001), and MAR (C17:0) was lower in LOV and VEG compared to OMN (*p* < 0.001). Similarly, POA (C16:1) was lower in VEG compared to OMN (*p* < 0.001) and the values were intermediate in LOV. In contrast, the other monounsaturated fatty acids OA (C18:1n9) and EA (C20:1n9) were higher in VEG, with significant differences compared to OMN and LOV in the placebo condition (both *p* < 0.001), while these differences were attenuated after the DHA-supplement. LA (C18:2n6) was significantly higher in VEG compared to OMN (*p* < 0.001) and ALA (C18:3n3) was higher in VEG and LOV than in OMN after the DHA-supplement and in VEG compared to OMN after the placebo. Serum GLA (C18:3n6) was also higher in VEG than in OMN, significantly after the DHA-supplement (*p* < 0.001), and DGLA (C20:3n6) was higher and AA (C20:4n6) lower in the two vegetarian groups (*p* = 0.005, *p* = 0.007, respectively). The values of serum EPA in vegetarians were about half of those of OMN participants (*p* < 0.001). All these serum fatty acids did not change by the DHA supplementation.

**Table 4:** Influence of DHA supplementation on serum fatty acids according to diet group (% of total fatty acids)

	Placebo			DHA-supplement			*p*		
	OMN	LOV	VEG	OMN	LOV	VEG	p_D_	p_T_	p_DxT_
MIR (C14:0)	0.65 ± 0.05	0.69 ± 0.06	0.60 ± 0.05	0.62 ± 0.04	0.67 ± 0.05	0.60 ± 0.08	0.170	0.600	0.818
PTC (C15:0)	0.20 ± 0.01	0.19 ± 0.01	0.10 ± 0.00^a, b^	0.19 ± 0.01	0.19 ± 0.01	0.10 ± 0.00^a, b^	** < 0.001**	0.734	0.939
PAL (C16:0)	20.98 ± 0.27	20.95 ± 0.25	19.22 ± 0.29^a, b^	21.21 ± 0.25	20.85 ± 0.39	19.16 ± 0.26^a, b^	** < 0.001**	0.962	0.685
POA (C16:1n7)	1.30 ± 0.08	1.15 ± 0.07	0.97 ± 0.06^a^	1.23 ± 0.07	1.09 ± 0.07	0.94 ± 0.06^a^	** < 0.001**	0.289	0.957
MAR (C17:0)	0.25 ± 0.01	0.22 ± 0.01^a^	0.17 ± 0.01^a^	0.25 ± 0.01	0.23 ± 0.01	0.18 ± 0.01^a^	** < 0.001**	0.573	0.190
STE (C18:0)	7.39 ± 0.12	7.55 ± 0.12	7.42 ± 0.15	7.55 ± 0.12	7.55 ± 0.16	7.54 ± .012	0.889	0.554	0.922
VAC(C18:1n7c)	1.54 ± 0.03	1.44 ± 0.04	1.45 ± 0.03	1.51 ± 0.03	1.49 ± 0.05	1.42 ± 0.03	**0.018**	0.781	0.402
OA (C18:1n9c)	22.06 ± 0.39	22.05 ± 0.54	23.94 ± 0.59^a, b^	22.30 ± 0.47	22.51 ± 0.70	23.50 ± 0.53	** < 0.001**	0.934	0.787
LA (C18:2n6c)	32.63 ± 0.61	34.05 ± 0.54	34.93 ± 0.48^a^	32.14 ± 0.53	33.90 ± 0.98	34.72 ± 0.59^a^	** < 0.001**	0.655	0.989
ALA (C18:3n3)	0.29 ± 0.02	0.34 ± 0.02	0.38 ± 0.03^a^	0.26 ± 0.02	0.37 ± 0.03^a^	0.40 ± 0.03^a^	** < 0.001**	0.842	0.565
GLA (C18:3n6)	0.38 ± 0.03	0.37 ± 0.03	0.46 ± 0.03	0.31 ± 0.02	0.34 ± 0.03	0.44 ± 0.04^a^	** < 0.001**	0.094	0.643
EA (C20:1n9)	0.21 ± 0.02	0.18 ± 0.01	0.26 ± 0.03^a, b^	0.19 ± 0.01	0.18 ± 0.01	0.23 ± 0.01^b^	** < 0.001**	0.138	0.155
EDA (C20:2n6)	0.20 ± 0.01	0.20 ± 0.01	0.22 ± 0.01	0.18 ± 0.01	0.19 ± 0.01	0.22 ± 0.01^a^	**0.007**	0.454	0.878
DGLA (C20:3n6)	1.54 ± 0.06	1.65 ± 0.07	1.72 ± 0.08	1.44 ± 0.05	1.53 ± 0.09	1.68 ± 0.08^a^	**0.005**	0.199	0.965
AA (C20:4n6)	7.42 ± 0.23	7.03 ± 0.33	6.75 ± 0.25	7.23 ± 0.20	6.47 ± 0.29	6.48 ± 0.28	**0.007**	0.100	0.810
EPA (C20:5n3)	0.49 ± 0.05	0.23 ± 0.02^a^	0.16 ± 0.02^a^	0.53 ± 0.06	0.25 ± 0.03^a^	0.28 ± 0.05^a^	** < 0.001**	0.116	0.615
ADA (C22:4n6)	0.21 ± 0.01	0.20 ± 0.01	0.23 ± 0.01	0.18 ± 0.01^c^	0.17 ± 0.01^c^	0.17 ± 0.01^c^	0.687	** < 0.001**	0.355
DPA (C22:5n3)	0.33 ± 0.01	0.27 ± 0.02	0.28 ± 0.02	0.28 ± 0.01^c^	0.19 ± 0.01^a, c^	0.22 ± 0.02^a, c^	** < 0.001**	** < 0.001**	0.743
DHA (C22:6n3)	1.94 ± 0.09	1.26 ± 0.08^a^	0.75 ± 0.05^a, b^	2.40 ± 0.10^c^	1.87 ± 0.07^a, c^	1.74 ± 0.07^a, c^	** < 0.001**	** < 0.001**	**0.018**
n-6	27.08 ± 2.10	43.49 ± 0.57^a^	44.31 ± 0.61^a^	38.09 ± 1.71	39.30 ± 2.38	42.24 ± 1.57	**0.004**	0.264	0.343
n-3	4.01 ± 2.52	3.75 ± 0.13	3.29 ± 0.12^a^	4.52 ± 0.23	3.89 ± 0.26	4.17 ± 0.21^c^	**0.021**	**0.008**	0.385
Ratio n-6/n-3	9.80 ± 0.52	11.98 ± 0.47^a^	13.93 ± 0.47^a, b^	8.75 ± 0.29^c^	10.43 ± 0.49^a, c^	10.47 ± 0.38^a, c^	** < 0.001**	** < 0.001**	**0.017**
DHA/AA	0.27 ± 0.01	0.19 ± 0.02^a^	0.12 ± 0.01^a, b^	0.34 ± 0.01^c^	0.30 ± 0.02^c^	0.28 ± 0.01^a, c^	** < 0.001**	** < 0.001**	**0.002**
AA + (EPA + DHA)	3.28 ± 0.15	5.03 ± 0.32^a^	8.22 ± 0.60ª^, b^	2.67 ± 0.12^c^	3.08 ± 0.14^a, c^	3.38 ± 0.15^a, c^	** < 0.001**	** < 0.001**	** < 0.001**
OA/STE	3.04 ± 0.09	2.95 ± 0.10	3.28 ± 0.11	3.00 ± 0.09	3.03 ± 0.13	3.15 ± 0.10	**0.048**	0.752	0.686
LA/OA	1.51 ± 0.04	1.58 ± 0.06	1.50 ± 0.05	1.48 ± 0.05	1.56 ± 0.08	1.51 ± 0.06	0.626	0.889	0.977
ALA/LA	0.01 ± 0.00	0.01 ± 0.00	0.01 ± 0.00	0.01 ± 0.00	0.01 ± 0.00^a^	0.01 ± 0.00^a^	** < 0.001**	0.794	0.547
LA/ALA	157.98 ± 28.91	106.85 ± 6.53	111.40 ± 9.13	155.47 ± 14.27	115.66 ± 14.69	95.01 ± 4.91^a^	** < 0.001**	0.909	0.288
GLA/LA	0.01 ± 0.00	0.01 ± 0.00	0.01 ± 0.00	0.01 ± 0.00	0.01 ± 0.00	0.01 ± 0.00	**0.019**	0.140	0.503
AA/DGLA	5.01 ± 0.20	4.47 ± 0.29	4.24 ± 0.28^a^	5.30 ± 0.26	4.54 ± 0.31	4.10 ± 0.25^a^	** < 0.001**	0.999	0.861
EPA/ALA	1.91 ± 0.22	0.68 ± 0.07^a^	0.42 ± 0.03^a^	2.34 ± 0.24	0.78 ± 0.09^a^	0.71 ± 0.11^a^	** < 0.001**	**0.016**	0.211
DHA/ALA	8.22 ± 0.72	4.04 ± 0.34^a^	2.39 ± 0.25^a, b^	11.61 ± 1.07^c^	6.65 ± 0.95^a, c^	4.75 ± 0.30^a, c^	** < 0.001**	** < 0.001**	0.171
DHA/EPA	5.34 ± 0.52	6.93 ± 0.72	5.99 ± 0.61	5.70 ± 0.34	9.76 ± 1.21^a^	8.26 ± 0.72^a, c^	**0.004**	**0.001**	0.136
EPA/AA	0.07 ± 0.01	0.03 ± 0.01^a^	0.02 ± 0.00^a^	0.08 ± 0.01	0.04 ± 0.00^a^	0.04 ± 0.01^a^	** < 0.001**	0.101	0.683
SFA	29.46 ± 0.30	29.59 ± 0.34	27.50 ± 0.32^a, b^	29.82 ± 0.26	29.48 ± 0.43	27.58 ± 0.31^a, b^	** < 0.001**	0.877	0.602
MUFA	25.11 ± 0.43	24.82 ± 0.56	26.62 ± 0.61^a^	25.22 ± 0.47	25.26 ± 0.73	26.09 ± 0.57	**0.017**	0.917	0.765
PUFA	45.42 ± 0.58	45.59 ± 0.53	45.88 ± 0.64	44.96 ± 0.53	45.26 ± 0.79	46.33 ± 0.61	0.304	0.897	0.819
PUFA/SFA	1.55 ± 0.03	1.55 ± 0.03	1.68 ± 0.03^a, b^	1.52 ± 0.03	1.55 ± 0.04	1.69 ± 0.03^a, b^	** < 0.001**	0.804	0.778
PUFA/MUFA	1.84 ± 0.05	1.87 ± 0.06	1.77 ± 0.06	1.83 ± 0.06	1.84 ± 0.08	1.81 ± 0.06	0.545	0.901	0.865
(PUFA + MUFA)/SFA	2.41 ± 0.03	2.39 ± 0.04	2.65 ± 0.04^a, b^	2.36 ± 0.03	2.41 ± 0.05	2.64 ± 0.04^a, b^	** < 0.001**	0.843	0.614

Values are expressed in mean ± SEM

Bold values indicate that the significant differences

*OMN* omnivorous, *LOV* lacto-ovo vegetarian, *VEG* vegan. Effects of diet group (D), treatment (T), and interaction (DxT), are presented. Within placebo or treatment

^a^Denotes significant differences compared to OMN

^b^Compared to LOV (at least *p* < 0.05). Between placebo and treatment

^c^Indicates significant differences compared to placebo from the same diet group (at least *p* < 0.05)

Regarding serum DHA, significant effects of diet, treatment and interaction diet x treatment were observed (Table 4). The values were higher in OMN followed by LOV and VEG (*p* < 0.001) after placebo. Consumption of the DHA-supplement increased DHA significantly compared to placebo in the three diet groups (*p* < 0.001), and it was observed a relatively higher increase in VEG that presented similar serum DHA than LOV after the DHA-supplement (*p* = 0.018, interaction diet x treatment). Nevertheless, LOV and VEG did not reach the DHA levels of OMN after the supplementation (Table 4). In contrast, the supplementation decreased ADA (C22:4n6) and DPA (C22:5n3) in the three diet groups (*p* < 0.001), the latter resulted lower in LOV and VEG compared to OMN.

Total n-6 shows significant effect of the diet (*p* = 0.004) but not of the supplementation. Values were higher in LOV and VEG than in OMN after placebo. Total n-3 shows that VEG had the lowest percentages after placebo (significantly compared to OMN) that increased by the supplementation (*p* = 0.008). The ratio n-6/n-3 was higher in VEG followed by LOV and OMN after placebo (*p* < 0.001) and significantly decreased in all groups after the DHA-supplement, although this ratio remained higher in LOV and VEG than in OMN (*p* < 0.001).

**Fig. 2:**
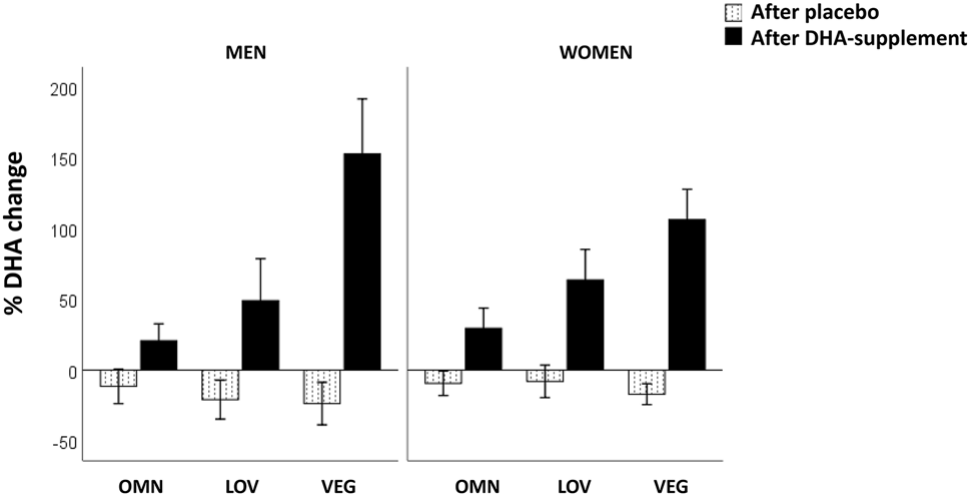
Change in serum DHA after intake the DHA-supplement or placebo, according to diet group and gender. *OMN* omnivorous, *LOV* lacto-ovo vegetarian, *VEG* vegan. All three diet groups were significantly different after DHA-supplement (*p* < 0.001) and there were no significant differences between diet groups after placebo. The differences between men and women were not significant (ANOVA)

To summarize, Fig. 2 shows the % change in serum DHA after consumption of the DHA-supplement compared to the placebo, according to diet group and gender. The increase in serum was not affected by gender, although women had higher DHA concentrations than men as indicated above, and there were significant differences due to diet (*p* < 0.001), with higher DHA elevations in the order VEG, 124 ± 10%; LOV 59 ± 8%; and OMN, 24 ± 4%.

## Discussion

This randomized controlled trial was performed in healthy volunteers who did not consume DHA or consumed very low amounts. Therefore, it was a vulnerable population that could benefit from its increased consumption and associated metabolic functions. The assayed DHA-supplement was manufactured from algae to be accepted by vegetarians who avoid animal products including fish, the principal dietary source of this fatty acid. The results clearly confirm that the tested supplement, at DHA dose of 250 mg/day, was effective in increasing serum % DHA in all three diet groups (OMN, LOV and VEG) which doubled in the case of vegans compared to placebo and baseline.

Volunteers were characterized by anthropometric values, body composition, blood pressure, heart rate, and serum glucose and lipids within normal reference ranges. Nutrient intakes showed the expected differences between vegetarians and non-vegetarians: the former ingested lower saturated fat and more carbohydrates than the latter. In addition, it was confirmed that DHA and EPA intakes were negligible in LOV and VEG and that all participants were correctly classified in one of the three diet groups.

Dietary intake did not vary during the trial and the DHA supplementation did not substantially change the differences between the three diet groups. Plant-based diets compared to omnivorous diets involve a higher consumption of PUFA at the expense of SFA, the contribution of which was mainly the essential LA (C18:2n6) [24], although the intake of the other essential fatty acid, ALA (C18:3n3), was also high in vegetarians as we observed throughout the assay (Table 3).

In the present study, results of dietary intake reveal that total fat intake was approximately 35–40% of the energy, as observed in several surveys performed in Spain [25, 26]. This has been related to the Mediterranean diet and the spread use of olive oil. Almost all our volunteers specified that they consumed olive oil as culinary fat and food ingredient. This fact was reflected on the estimation of OA intake that contributed to the highest dietary fatty acid, approximately 25–30 g/day, with moderate differences between diet groups (Table 2).

Intake of LA was on average 4.3, 5.0 and 5.5% of total energy (%E), while ALA was 0.6, 0.7, and 0.6%E, in OMN, LOV and VEG, respectively. Considering that LA intake is relatively high in Europe and that the lowest average intake is not associated with deficiency, the European Food Safety Authority (EFSA) proposed an Adequate Intake for n-6 PUFA of 4 E% and for ALA of 0.5 E% without risk of deficiencies [27]. Therefore, results of LA and ALA intake of the present study indicate that volunteers ingested sufficient amounts of both essential fatty acids. However, the proposed reference n-6/n-3 ratio below 10:1 [28] was at the upper level in LOV and above in vegans who ingested more LA compared to the other groups and whose n-3 intake was almost exclusively ALA (Tables 2 and 3). This is in line with findings indicating that the abundance of LA in the diet reduces the transformation of ALA into their long chain fatty acid derivatives, EPA and DHA [8, 29]. Therefore, although vegans ingested enough ALA its metabolism could be inhibited in a certain extent, due to interaction between the n-3 and n-6 pathways, as explained below.

In omnivorous, it was estimated an average intake of DHA, at baseline or with placebo, of 440–500 mg/day, and a EPA intake nearly 200 mg/day, which is above the recommended intake of 250 mg for DHA alone or 250–500 mg/day for EPA + DHA [10–12]. However, this was probably overestimated, as these volunteers had to be low consumers of fish to be selected for the trial (maximum two times per week). Lower values of EPA and DHA intakes have been reported in an exploratory study in a small group of Australian omnivorous and vegan endurance athletes [30]. In any case, these results also show that the dose of DHA in the tested algae supplement was at the lowest threshold of what was consumed by the average omnivorous participant, whose maximum intake was two servings of fish per week.

Overall, the dietary intake results support that the usual diet of the volunteers did not change during the assay, and an imbalance in fatty acids intake was observed, with a relative excess of the n-6 LA in detriment of the two n-3, EPA and DHA, which were absent in the diet of vegans, who just depend on the metabolic synthesis of these long-chain fatty acids.

Results of serum percentages of fatty acids show that the major fatty acid was LA (C18:2n6) and that it remained similar after consumption of the DHA-supplement without differences due to treatment. Vegans presented the highest %LA values either after the placebo or the DHA-supplement (Table 4). Likewise, as for the second most abundant fatty acid in serum, OA (C18:1, n9), there was no effect of treatment. This should be related to the predominant use of olive oil by the participants, independently of their diet classification, and with the composition of the DHA-supplement that did not include OA. Consumption of the placebo capsules did not further increase serum %OA from baseline as in fact the intake of OA from the placebo represented only a small fraction of the total dietary intake of this fatty acid. It should be noticed that fatty acid profiles of the present work are consistent but slightly differ from the global fatty acid database reported by Brenna [31] (see also supplemental Table S3). In the present Spanish study, the percentage of OA in serum resulted higher than of palmitic acid (C16:0), that was the second more abundant fatty acid in the mentioned report which primarily collected data from Nord American laboratories. Furthermore, our EPA and DHA data were relatively low and should be used as a reference for low-fish consumers and vegetarians living in a Mediterranean country.

In addition, serum fatty acids confirm that there was some endogenous production of EPA and DHA in vegetarians (Table 4). Although the diets LOV and VEG did not provide appreciable amounts, both fatty acids were detected in serum, and this should be attributed to biosynthesis from the common precursor ALA (C18:3n3). Interestingly, serum DHA was much higher in women than in men and this difference was unaffected by dietary classification or DHA-supplement. The literature consistently reports that this is due to hormonal differences, probably related to reproduction and infant protection since DHA is crucial for brain development [32].

**Fig.3:**
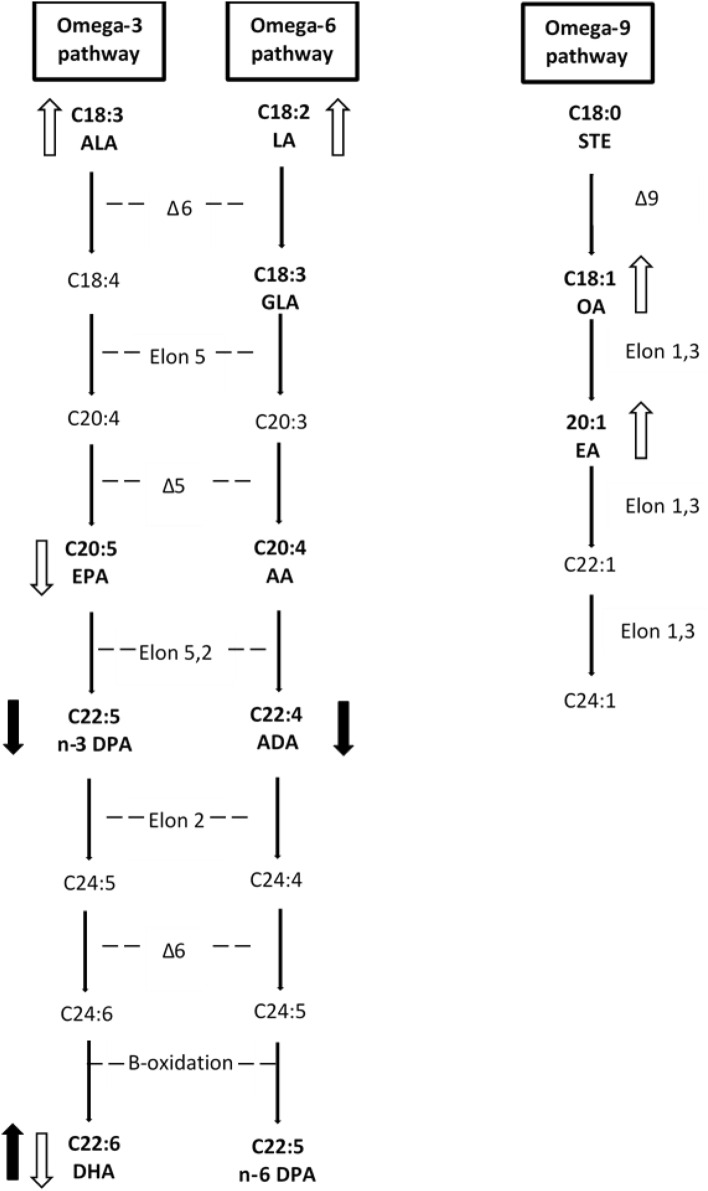
Biosynthesis of long chain polyunsaturated fatty acids in humans and changes due to vegetarian diets and DHA supplementation of the present randomized controlled trial. Fatty acids analyzed in this study are in bold. Δ6: desaturase Δ6; Δ5: desaturase Δ5; Δ: desaturase Δ9; Elon: elongase. Differences due to diet type: open arrows indicate significantly increased or decreased in lacto-ovo vegetarians and/or vegans compared to omnivorous. Changes due to the DHA supplementation: dark arrows indicate significant increase or decrease after consumption of the DHA-supplement in the three diet groups

However, the metabolic efficiency of the n-3 pathway could be partly inhibited in vegetarians. It is known that there is a relationship between the n-3 and n-6 fatty acid pathways, because there is a competition for the same enzymes, desaturases and elongases, that participate in the synthesis of the long-chain fatty acids of the two families. The rate limiting enzyme is the Δ-6 desaturase that has higher affinity for ALA than LA. However, under conditions of high LA intake as in the present study, the n-6 pathway predominates over the n-3 pathway. As shown in Fig. 3, EPA (C20:5n3) and AA (C20:4n6) result from sequential desaturation and elongation steps using the same enzymes. These fatty acids can be converted to metabolic mediators or continue through the n-3 and n-6 cascades to yield C24:6n3 and C24:5n6, then transported to peroxisomes for beta-oxidation, and finally produce DHA (C22:6n3) and docosapentaenoic acid (C22:5n6) [33, 34]. The conversion rate of ALA to DHA has been estimated to be lower than 1% or as low as 0.01%, depending on the used models [5, 35].

In this regard, it was obtained that vegetarians presented lower serum EPA and DHA than omnivorous as expected, and at the same time, they had higher %ALA than omnivorous, which was particularly shown in vegans. However, because their serum LA was elevated, which was clearly seen in vegans, our results support that the conversion of ALA into EPA and DHA was very limited, especially in this diet group, due to the imbalance n-6/n-3 in addition to the virtually no DHA intake. This is in accordance with our previous study comparing LOV and VEG in which we obtained even more elevated serum n-6/n-3 ratios, indicative of metabolic imbalance [8]. This low metabolic production of DHA was estimated by the DHA/ALA ratio that was lower in VEG and LOV compared to OMN after placebo, with marked differences between the three diet groups, suggesting that the biosynthesis of DHA form its precursor was compromised more in vegans than in the other vegetarian group. However, these estimations in terms of ratios do not give information on the relative content in tissues, such as red cell membranes and heart, and much more research is needed in animal models and humans to know the effects of varying n-3 and n-6 fatty acid intakes into tissue levels and biological function [29, 36].

Concerning the influence of the intervention with the DHA-supplement on serum fatty acids, it was clearly efficacious in increasing serum DHA, independently of the diet of the volunteers, did not modify serum total n-6 fatty acids or saturated fatty acids, and improved the ratio n-6/n-3, specially in vegans. Stark et al. [37, 38] calculated EPA + DHA in erythrocytes (omega-3 index, O3I) from the values of EPA + DHA in different blood fractions, including total plasma/serum, to unify data and identify countries potentially at an increased risk of chronic disease. We have estimated the O3I from our data in serum and we have obtained that serum EPA + DHA is approximately 2% in omnivorous after placebo and ≥ 3% after the DHA supplementation. These values are equivalent to the lowest erythrocyte O3I category of ≤ 4% given by these authors indicative of low cardiovascular protection. The LOV and VEG diet groups had serum EPA + DHA of 1–1.5% with placebo and approximately 2% after the DHA-supplement. This means that all our participants, including the diet group OMN and even after consuming the DHA-supplement, would be in the lowest category of O3I which would imply the highest disease risk. However, we are aware of the limitations of this estimation and our results are far from supporting a recommendation in terms of cardiovascular risk.

Figure 2 shows that the DHA increment in serum was much higher in VEG, followed by LOV and OMN, and it is important to note that DHA significantly increased in the three diet groups. Moreover, it was observed that the values increased similarly in men and women who presented generally higher DHA concentrations than men. Therefore, these results lead us to the conclusion that all participants with low or null intake of DHA would benefit from the consumption of the tested DHA-supplement. Other attempts to improve the efficiency of the n-3 pathway, such as reducing LA intake and increasing that of ALA, for example by consumption an ALA supplement, were not able to increase DHA levels [5, 29, 35, 39].

Nevertheless, our results show that serum DPA (C22:5 n3) and ADA (C22:4 n6) decreased by the DHA supplementation. Our results are consistent with others who also found lower DPAn3 levels after DHA supplementation [14, 17, 40]. To explain this result, on one hand, it could be argued that the n-3 pathway is partly inhibited with the DHA supplementation, as abundance of the product inhibits its own synthesis (Fig. 3). Our results suggest that the n-3 pathway slows down at the EPA level as the immediate metabolite DPA is markedly decreased, probably because the elongase-2 activity is reduced. On the other hand, it has been suggested that this is likely due to increased competition for incorporation into complex lipids by acyltransferases due to higher amounts of free DHA [14, 17]. However, the exact mechanism is unknown and this deserves further investigation.

In support to this metabolic change, we observed that both DPA (n-3) and ADA (n-6) decline and that the ratio EPA/ALA increases after the DHA supplementation. In agreement, we previously observed that vegetarians who consumed n-3 supplements presented increases in serum DHA and also in EPA, which initially we had associated to the composition of some supplements made from fish oil that they ingested [8]. Other authors have reported an increase in circulating EPA after DHA supplementation, but in their studies, the DHA dose was higher than in the present study. This opened the possibility of a retroconversion of DHA to EPA [14, 15, 17], although this has not been demonstrated and it is more likely that EPA is spared from further metabolism to DHA [40, 41].

Since our design includes the gender effect, we observed that women presented higher serum DHA than men. This agrees with reports suggesting that a sexual dimorphism leads to higher serum DHA in women than in men. Women may have a greater capacity for DHA synthesis from ALA than men, which has been attributed to oestrogens [32]. However, both genders responded with similar elevations of serum DHA after supplementation (Fig. 2).

Figure 3 summarises the main findings of the current study. Serum long chain fatty acids vary due to the usual diet and the DHA supplementation. Vegetarian diets compared to omnivore diets involve higher serum values of the two precursors of the n-3 and n-6 pathways (ALA and LA) but lower n-3 EPA and DHA. This was particularly shown with the vegan diet that in addition leads to higher n-9 fatty acids, OA and EA, than the other type of diets. The tested DHA-supplement compared to placebo produces a marked increase in serum DHA, independently of usual diet and sex, a decrease in the two intermediate metabolites DPAn3 and ADAn6, and appears to spare EPA for metabolic functions apart from that of DHA synthesis.

This crossover randomized controlled trial was the first to investigate the bioavailability of 250 mg of a new algal DHA-supplement in lacto-ovo vegetarians, omnivorous and vegan volunteers. Some studies found significant increases in plasma DHA levels with higher doses than our supplement had and during shorter time of intervention [22, 42, 43]. We observed that ingestion of one capsule containing of 250 mg of DHA during 5 weeks was acceptable by the volunteers without discomfort, was compatible with the usual diet, and delivered highly bioavailable DHA. Moreover, reports on intakes of fatty acids and circulating levels of vegetarian compared with omnivorous populations are very scarce and lacking in Spain.

The strengths of the study were: the sample size planned was achieved and was sufficient to detect very significant effects. The three diet groups, lacto-ovo vegetarian, vegan and omnivore, were characterized and the differences between them were obtained with very important differences in nutrient intakes and serum fatty acids. The crossover randomised controlled trial was successfully performed and there were no carry-over effects. Moreover, the gender effect was also observed but women and men responded similarly to the DHA-supplement. It was confirmed that the placebo made of olive oil resembled the main fat of the usual diet, either omnivorous or vegetarian, of the volunteers. In addition, there are very few studies with a moderate amount of supplement, and the dose used on this one is the recommended for this type of supplement.

There are some limitations of this study: analyses were performed in serum and there is no information on membrane fatty acids or tissue concentrations. However, since our objective was to know the bioavailability of DHA from the algae source, we were aware of this limitation from the beginning. In addition, we did not study any DHA function. Finally, the genetic factor associated to fatty acid metabolism was not taken into account.

Present results clearly demonstrate that the supplement of DHA from the microalgae *Schizochytrium sp*. was bioavailable and useful for raising circulating levels of this important n-3 long-chain fatty acid. This novel DHA-supplement has a great potential, as a variety of consumers of plant-based diets, could benefit from it. Innovation in this field has been reviewed in detail [44]. Furthermore, among omnivorous, those who avoid fish or should not ingest fish products, due to pathologies such as allergy to *Anysakis sp*. for example could also benefit from its consumption, as well as the general population who is open to new formulations, supplements and functional foods.

## Supplementary Information

Below is the link to the electronic supplementary material.

Supplementary file1 (DOCX 19 KB)

## Data Availability

The data can be shared by request.

## Abbreviations

MIR (C14:0): Myristic acid
PTC (C15:0): Pentadecylic acid
PAL (C16:0): Palmitic acid
POA (C16:1N7): Palmitoleic acid
MAR (C17:0): Margaric acid
STE (C18:0): Stearic acid
VAC (C18:1N7): Vaccenic acid
OA (C18:1N9): Oleic acid
LA (C18:2N6): Linoleic acid
ALA (C18:3N3): α-Linolenic acid
GLA (C18:3N6): γ-Linolenic acid
EA (C20:1N9): Gondoic acid
EDA (C20:2N6): Eicosadienoic acid
DGLA (C20:3N6): Dihomo-γ-Linolenic acid
AA (C20:4N6): Arachidonic acid
EPA (C20:5N3): Eicosapentaenoic acid
ADA (C22:4N6): Docosatetraenoic acid
DPA (C22:5N3): Docosapentaenoic acid
DHA (C22:6N3): Docosahexaenoic acid
n-6: Omega-6 fatty acids
n-3: Omega-3 fatty acids
SFA: Saturated fatty acids
MUFA: Monounsaturated acids
PUFA: Polyunsaturated fatty acids
BMI: Body mass index
HDL: High-density lipoprotein
LDL: Low-density lipoprotein
OMN: Omnivorous
LOV: Lacto-ovo vegetarian
VEG: Vegan
