# Supplementary material for: Changes in fatty acid levels after consumption of a novel docosahexaenoic supplement from algae: a crossover randomized controlled trial in omnivorous, lacto-ovo vegetarians and vegans
Source: Eur J Nutr. 2022 Nov 23;62(4):1691–705. doi: 10.1007/s00394-022-03050-3 (PMC9684969; doi:10.1007/s00394-022-03050-3)
Supplement: Supplementary file 1 — Supplementary file1 (DOCX 19 KB) [file 394_2022_3050_MOESM1_ESM.docx]

| **Fatty acid** | **% area GC** | | **mg/g as FA** |
| --- | --- | --- | --- |
| C12:0 | 0.8 |  | |
| C14:0 | 9.2 |  | |
| C16:0 | 22.5 |  | |
| C16:1 n7 | 2.9 |  | |
| C18:0 | 0.8 |  | |
| C18:1 n9c | 2.3 |  | |
| C18:1 n7c | 4.4 |  | |
| C18:2 n6c | 1.1 |  | |
| C20:4 n3 | 0.5 |  | |
| C20:5n-3 | 1.6 | | 15 |
| C22:5 n6 | 8.0 |  | |
| C22:5 n3 | 0.3 |  | |
| C22:6n3 | 44.9 | | 425 |
| Total Omega-3* | 47.3 | | 450 |

**Supplementary**

**Table S1** *Schizochytrium sp* oil composition contained in the DHA capsules.

**Table S2** Olive oil composition contained in the placebo capsules

| **Fatty acid** | **%** |
| --- | --- |
| C14:0 | \| 0.01 \| \| --- \| |
| C16:0 | 10.62 |
| C16:1 | 0.85 |
| C17:0 | 0.07 |
| C17:1 | 0.11 |
| C18:0 | 3.25 |
| C18:1 | 77.39 |
| C18:2 | 6.15 |
| C18:3 | 0.62 |
| C20:0 | 0.40 |
| C20:1 | 0.25 |
| C22:0 | 0.12 |
| C24:0 | 0.06 |
| C18:1T | 0.05 |
| C18:2T + C18:3T | 0.06 |

**Table S3** Percentiles of serum fatty acid at baseline (% of total fatty acids)

|  | **OMN** | | | | | **LOV** | | | | | | | **VEG** | | | | | | |
| --- | --- | --- | --- | --- | --- | --- | --- | --- | --- | --- | --- | --- | --- | --- | --- | --- | --- | --- | --- |
|  | **5** | **25** | **50** | **75** | **95** | **5** | **25** | **50** | | **75** | | **95** | **5** | **25** | | **50** | | **75** | **95** |
| MIR (C14:0) | 0.34 | 0.44 | 0.55 | 0.74 | 1.37 | 0.30 | 0.44 | | 0.65 | | 0.89 | 1.53 | 0.28 | | 0.38 | | 0.45 | 0.64 | 1.07 |
| PTC (C15:0) | 0.10 | 0.15 | 0.18 | 0.22 | 0.32 | 0.07 | 0.14 | | 0.19 | | 0.24 | 0.31 | 0.05 | | 0.07 | | 0.09 | 0.11 | 0.35 |
| PAL (C16:0) | 18.18 | 19.66 | 20.89 | 21.92 | 23.49 | 17.46 | 19.39 | | 20.33 | | 21.26 | 23.43 | 15.41 | | 17.34 | | 18.11 | 19.85 | 21.64 |
| POA (C16:1n7) | 0.71 | 0.98 | 1.18 | 1.45 | 2.36 | 0.61 | 0.98 | | 1.26 | | 1.47 | 1.94 | 0.57 | | 0.76 | | 0.92 | 1.15 | 1.72 |
| MAR (C17:0) | 0.18 | 0.21 | 0.23 | 0.26 | 0.29 | 0.15 | 0.18 | | 0.22 | | 0.25 | 0.28 | 0.13 | | 0.15 | | 0.16 | 0.19 | 0.23 |
| STE (C18:0) | 5.97 | 6.72 | 7.30 | 7.88 | 8.72 | 6.01 | 6.92 | | 7.35 | | 7.91 | 8.29 | 5.74 | | 6.67 | | 7.21 | 7.59 | 8.38 |
| VAC (C18:1n7) | 1.21 | 1.35 | 1.56 | 1.69 | 1.8 | 1.04 | 1.25 | | 1.41 | | 1.61 | 1.91 | 1.18 | | 1.33 | | 1.50 | 1.61 | 1.92 |
| OA (C18:1n9c) | 18.46 | 20.40 | 21.46 | 23.14 | 25.86 | 17.16 | 19.41 | | 21.69 | | 23.48 | 26.59 | 18.87 | | 22.11 | | 23.46 | 25.68 | 32.50 |
| LA (C18:2n6c) | 28.12 | 30.20 | 32.32 | 33.95 | 36.78 | 28.04 | 31.90 | | 34.84 | | 37.00 | 45.82 | 26.71 | | 32.16 | | 34.60 | 38.30 | 41.19 |
| ALA (C18:3n3) | 0.13 | 0.19 | 0.23 | 0.29 | 0.51 | 0.20 | 0.26 | | 0.31 | | 0.43 | 0.66 | 0.23 | | 0.31 | | 0.39 | 0.55 | 0.79 |
| GLA (C18:3n6) | 0.13 | 0.25 | 0.35 | 0.46 | 0.63 | 0.08 | 0.29 | | 0.45 | | 0.52 | 0.86 | 0.21 | | 0.38 | | 0.43 | 0.51 | 0.79 |
| EA (C20:1n9) | 0.12 | 0.15 | 0.18 | 0.21 | 0.28 | 0.10 | 0.16 | | 0.18 | | 0.22 | 0.30 | 0.14 | | 0.20 | | 0.26 | 0.32 | 0.54 |
| EDA (C20:2n6) | 0.17 | 0.20 | 0.23 | 0.26 | 0.33 | 0.14 | 0.21 | | 0.23 | | 0.28 | 0.39 | 0.18 | | 0.25 | | 0.28 | 0.33 | 0.51 |
| DGLA (C20:3n6) | 0.87 | 1.24 | 1.54 | 1.8 | 2.43 | 0.82 | 1.38 | | 1.57 | | 1.95 | 2.30 | 0.44 | | 1.45 | | 1.63 | 2.07 | 2.92 |
| AA (C20:4n6) | 5.75 | 6.96 | 8.00 | 8.94 | 10.85 | 3.20 | 6.41 | | 7.18 | | 8.10 | 9.07 | 4.72 | | 5.62 | | 7.32 | 8.13 | 10.40 |
| EPA (C20:5n3) | 0.15 | 0.31 | 0.46 | 0.64 | 1.28 | 0.04 | 0.15 | | 0.19 | | 0.23 | 0.39 | 0.08 | | 0.13 | | 0.19 | 0.26 | 0.33 |
| ADA (C22:4n6) | 0.10 | 0.18 | 0.24 | 0.30 | 0.43 | 0.13 | 0.21 | | 0.28 | | 0.31 | 0.40 | 0.11 | | 0.21 | | 0.29 | 0.32 | 0.39 |
| DPA (C22:5n3) | 0.21 | 0.25 | 0.31 | 0.36 | 0.58 | 0.12 | 0.20 | | 0.23 | | 0.29 | 0.39 | 0.08 | | 0.18 | | 0.26 | 0.33 | 0.47 |
| DHA (C22:6n3) | 1.24 | 1.90 | 2.2 | 2.59 | 3.37 | 0.67 | 1.12 | | 1.25 | | 1.33 | 2.12 | 0.54 | | 0.64 | | 0.78 | 0.99 | 1.37 |
| Ratio n-6/n-3 | 5.80 | 7.85 | 8.85 | 10.00 | 13.27 | 8.46 | 10.74 | | 11.51 | | 13.57 | 20.27 | 9.54 | | 11.64 | | 12.95 | 15.07 | 21.19 |
| DHA/AA | 0.16 | 0.22 | 0.28 | 0.33 | 0.44 | 0.12 | 0.14 | | 0.17 | | 0.21 | 0.42 | 0.07 | | 0.09 | | 0.11 | 0.13 | 0.20 |
| AA+(EPA+DHA) | 1.57 | 2.47 | 2.98 | 3.94 | 5.53 | 2.23 | 4.26 | | 4.81 | | 5.94 | 6.62 | 3.99 | | 5.86 | | 7.45 | 8.54 | 10.19 |
| OA/STE | 2.31 | 2.67 | 3.08 | 3.34 | 4.14 | 2.33 | 2.57 | | 2.91 | | 3.31 | 3.88 | 2.39 | | 2.89 | | 3.40 | 3.84 | 4.99 |
| LA/OA | 1.18 | 1.36 | 1.47 | 1.62 | 1.88 | 1.12 | 1.38 | | 1.63 | | 1.87 | 2.55 | 0.86 | | 1.27 | | 1.45 | 1.70 | 2.19 |
| ALA/LA | 0.00 | 0.01 | 0.01 | 0.01 | 0.016 | 0.01 | 0.01 | | 0.01 | | 0.01 | 0.02 | 0.01 | | 0.01 | | 0.01 | 0.02 | 0.02 |
| GLA/LA | 0.00 | 0.01 | 0.02 | 0.01 | 0.02 | 0.00 | 0.01 | | 0.01 | | 0.02 | 0.03 | 0.01 | | 0.01 | | 0.01 | 0.01 | 0.03 |
| AA/DGLA | 3.34 | 4.37 | 5.29 | 6.34 | 8.32 | 2.75 | 3.70 | | 4.14 | | 5.05 | 5.99 | 1.69 | | 3.26 | | 4.05 | 5.59 | 23.77 |
| EPA/ALA | 0.70 | 1.27 | 2.03 | 2.93 | 4.98 | 0.13 | 0.42 | | 0.56 | | 0.80 | 1.28 | 0.21 | | 0.34 | | 0.46 | 0.60 | 0.73 |
| DHA/ALA | 3.60 | 8.01 | 9.86 | 11.24 | 17.03 | 1.15 | 2.74 | | 4.02 | | 5.16 | 8.58 | 0.83 | | 1.46 | | 1.91 | 2.89 | 4.53 |
| DHA/EPA | 2.19 | 3.37 | 4.71 | 6.60 | 11.19 | 3.01 | 4.55 | | 6.25 | | 11.38 | 27.74 | 2.28 | | 2.91 | | 4.27 | 7.39 | 12.81 |
| EPA/AA | 0.02 | 0.04 | 0.06 | 0.09 | 0.20 | 0.01 | 0.02 | | 0.03 | | 0.038 | 0.05 | 0.01 | | 0.02 | | 0.03 | 0.04 | 0.05 |
| SFA total | 26.54 | 28.13 | 29.07 | 29.81 | 32.12 | 24.89 | 28.07 | | 28.74 | | 29.95 | 32.77 | 23.15 | | 25.39 | | 26.33 | 27.82 | 29.20 |
| MUFA total | 21.26 | 23.30 | 24.77 | 26.09 | 28.69 | 19.83 | 21.94 | | 24.93 | | 26.40 | 29.03 | 21.19 | | 24.87 | | 26.49 | 28.33 | 35.58 |
| PUFA total | 42.37 | 44.80 | 46.06 | 47.42 | 49.75 | 40.86 | 43.87 | | 46.41 | | 48.34 | 53.75 | 37.35 | | 44.32 | | 47.30 | 49.42 | 53.24 |
| PUFA/SFA | 1.36 | 1.51 | 1.60 | 1.67 | 1.86 | 1.28 | 1.52 | | 1.60 | | 1.73 | 2.10 | 1.38 | | 1.61 | | 1.78 | 1.92 | 2.16 |
| PUFA/MUFA | 1.54 | 1.72 | 1.87 | 2.03 | 2.37 | 1.46 | 1.68 | | 1.84 | | 2.16 | 2.65 | 1.04 | | 1.56 | | 1.77 | 2.00 | 2.50 |
| (PUFA+MUFA) /SFA | 2.11 | 2.35 | 2.44 | 2.56 | 2.77 | 2.0515 | 2.33 | | 2.48 | | 2.56 | 3.02 | 2.42 | | 2.60 | | 2.80 | 2.94 | 3.32 |
